# Alleviating Pressure on Water Resources: A new approach could be attempted

**DOI:** 10.1038/srep14006

**Published:** 2015-09-14

**Authors:** Shikun Sun, Yubao Wang, Feifei Wang, Jing Liu, Xiaobo Luan, Xiaolei Li, Tianwa Zhou, Pute Wu

**Affiliations:** 1 College of Water Resources and Architectural Engineering, Northwest A&F University, Yangling, 712100, China; 2 Institute of Water Saving Agriculture in Arid regions of China, Northwest A&F University, Yangling, 712100, China; 3 Key Laboratory of Agriculture al Soil and Water Engineering in Arid and Semiarid Areas, Ministry of Education, Northwest A&F University, Yangling, 712100, China; 4 College of Economics and Management, Northwest A&F University, Yangling, 712100, China; 5 Institute of Soil and Water Conservation, Northwest A&F University, Yangling, 712100, China; 6 Institute of Soil and Water Conservation, Chinese Academy of Sciences and Ministry of Water Resources, Yangling, 712100, China

## Abstract

Water and food safety are two major challenges which the world faces today. Traditional water management focuses on the reduction of water use through improvements in water saving technologies. However, quantitative research is needed to evaluate the effects of changing food consumption patterns on water resources. Here we report the water saving effects of changing diet pattern of the major crops and animal products in mainland China. By using the concepts of water footprint (WF) per weight unit and per calorie unit, provided by 13 primary crop and animal products, the WFs of the 13 agricultural products in each province are compared, and their water/energy conversion efficiencies are analyzed. Then, impacts of different scenarios of changing diet pattern on water consumption were explored. Results show that there are obvious differences between the WF per weight and calorie unit provided by crop and animal products due to the nutritional properties of the agricultural products. Promoting water savings from the food consumption side could give a positive feedback on water consumption. Scenario analysis of adjustments to the diet pattern proves that it is potentially feasible to reach the objective of alleviating stress on water resources while guaranteeing nutritional value of the residents.

Limited water resources and food safety are one of the main challenges the world will face in the future. By 2030, the world population is predicted to increase beyond 8 billion, which will inevitably put great pressure on the food and water resources of the planet^1^,^2^. As one of the major population and agricultural countries of the world, China will feel this pressure as well. Not only will water resources be stressed from an increasing population and food demands, there will be increasing competition from industry, in addition to the impacts of climate change^3^. While improving crop production is necessary for future food needs, the limited water resources will not be able to meet the necessary water requirements, and China must therefore increase its food production with the same, or even less, amount of agricultural water than is uses today^4^. Getting more crops per drop, particularly in areas where water could become scarcer due to climate change, will be essential to achieve food security worldwide, especially for China^5^. The traditional techniques for agricultural water management seek improvements in crop water productivity through methodologies in agriculture, biology and engineering; such as promoting water saving irrigation technology (sprinkler irrigation and micro irritation), adopting plastic filled mulching to reduce the evaporation of the soil, and increasing the drought tolerance of a crop through selective breeding or genetic modifications^6^,^7^,^8^. Thus, traditional water resource management primarily focuses on water distribution and increasing water productivity during the production process. While, the quantitative analysis research on a possible reduction of water consumption in agriculture production through changing of the dietary pattern in the consumer population would provide a new strategy to alleviate the water stress.

An effective way to discuss the significance of consumption patterns on water usage is through the water footprint concept. The water footprint is an indicator which expresses the multiple effects that human production and consumption have on water resources and environment, from the water consumption type, quantity, purpose and efficiency^9^,^10^. The concept of a water footprint serves as a new indicator when analyzing the relationship between water resources and consumption needs, and provides a basis for decisions about proper water usage. The traditional evaluation system of managing production resources can now be extended to include product consumption management which could be a method for improving the efficiency of water use^11^. Currently, research conducted on water footprints primarily focuses on three aspects: 1) water footprint theory and quantitative analysis, 2) case studies, and 3) water resource evaluations and management based on water footprint. Zhao *et al.* (2009)^12^ presented a framework for calculating the national water footprint with input–output methods on China for 2002. Hubacek *et al.* (2009) explored the current trajectories and scenarios for urbanization and lifestyle changes and other important socio-economic trends in China by using Ecological and Water Footprints. The study indicated that the ability to construct sustainable communities in the future is a key challenge for China. Hoekstra *et al.* (2011)^10^ proposed the water footprint quantitative analysis and evaluation framework, and elaborated on the objective and strategies of the water footprint. Fader *et al.* (2011)^13^ quantified both the green and the blue internal and external WFPs of countries for major crop types in the world based on a dynamic global vegetation and water balance model. Chapagain and Tickner (2012)^14^ pointed out that water footprint is an effective tool to evaluate water consumption and could contribute to better understanding of the connections between water use, economic development, business practice and social and environmental risks. Water resource evaluation and management studies include Zhang *et al.* (2013)^15^ who pointed out that the Water Footprint Assessment provides a new solution for determining the effects that human consumption can have on water resources, thus helping management organizations to achieve sustainable development.

With the increasing demand for food and escalating water shortages in many parts of the world at the same time, researchers began to analyze the impact of food consumption patterns on water resources consumption^16^,^17^,^18^,^19^,^20^,^21^,^22^,^23^. Liu *et al.* (2008)^16^ quantified how food consumption patterns influence water requirements in China, and concluded that the effect of the food consumption patterns on China’s water resources is substantial. Mekonnen and Hoekstra (2012)^17^ assessed the water footprint of farm animals and of the various derived animal products per country and per animal production system. Results showed it is more water-efficient to obtain calories through crop products than animal products. Vanham *et al.* (2013)^18^ quantified the water footprint of consumption related to agricultural products for three different diet patterns in EU, and they found that a lot of water could be saved through dietary changes. Ercin and Hoekstra (2014)^19^ analyzed global water consumption and simulated the WF of food production under the conditions of future population and economic growth, along with changes in the dietary consumption of the population. Hess *et al.* (2015)^20^ considered the spatially explicit potential impact of alternative, healthier eating scenarios for the UK on global blue water scarcity by using the concept of a water scarcity footprint. The above studies provided a new field of vision on water management.

As the most populous country in the world, food security in China has attracted much concern around the world. A comprehensive assessment of regional water productivity of primary crop and animal products in China is needed, as it can provide the information for government institutions and the general public to develop more water-efficient dietary patterns. This article uses the concept of water footprint and extends its base by proposing the concept of WF per calorie provided by an agricultural product. Differences in WF per weight and calorie between provinces and agricultural products were discussed, as well as the water/energy conversion efficiencies for the main agricultural products in mainland China. Then, the potential of water saving through changes in diets pattern was assessed. The results of this study can serve as a framework to a water-saving dietary plan and as a potential solution to future water shortages in agricultural production.

## Data and Method

### The data related to this research included the following

#### Agricultural Data

The data for crop production, consumption, sown area, crop yield and agricultural inputs are from the Chinese statistical year book (2011)^24^ and China Agriculture Statistical Report (2011)^25^.

#### Dietary pattern scenarios setting

**Figure 1:**
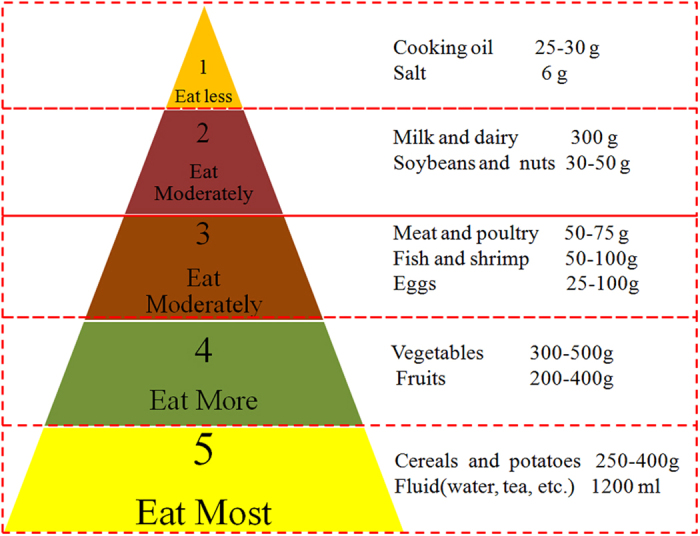
Daily dietary Guidelines for Chinese Residents (Revised from CNS, 2013).

In the water savings through changes in diets section, the recommended daily dietary pattern was referenced from the ‘Dietary Guidelines for Chinese Residents 2011′ from the Chinese Nutrition Society (CNS)^26^ (Fig. 1).

#### Water footprint of the agricultural products

The data related to water footprint of major *agricultural* products in China were taken from the results of the study by Mekonnen and Hoekstra (2011)^27^ and Mekonnen and Hoekstra (2010)^28^.

### Quantitative method of water footprint

The water footprint per unit weight of crop is formed by three parts: blue water footprint (the underground and surface water consumed through evapotranspiration during crop growth), green water footprint (the precipitation consumed through evapotranspiration during crop growth period), and grey water footprint (the fresh water needed for the diluting the fertilizer and pesticides used to environmentally safe concentrations). The quantitative method of water footprint per unit weight of crop proposed by Hoekstra *et al.*^10^ is as follows:


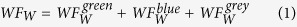


Where 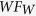 is the water footprint per weight unit of the crop in m^3^/kg; 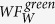 is the green water footprint in m^3^/kg; 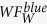 is the blue water footprint in m^3^/kg; 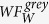 is the grey water footprint in m^3^/kg. And the detail calculation process refer to Hoekstra *et al.* (2011)^10^

Water footprint of animal products can be calculated as follows^17^:


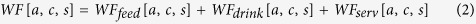


where 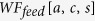, 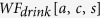 and 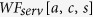 represent the water footprint of an animal for animal category *a* in country *c* in production systems *s* related to feed, drinking water and service-water consumption, respectively, all in m^3^.

The calculation of the water footprint per calorie unit provided by the agricultural products is as follows:


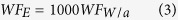


Where 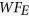 is the water footprint per calorie provided by the agricultural product, L/kcal; 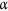 is the energy provided per weight unit of the crop, kcal/kg; 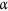 for the crop and animal products in this article are based on the related data provided by the Food and Agriculture Organization(FAO)^16^,^29^.

### Potential water saving calculation

The volume of water savings through changes in the dietary consumption pattern can be calculated as follows:


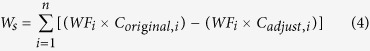


Where the 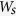 is the volume of water savings through changes in the dietary consumption pattern, m^3^; 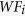 is the water footprint of product 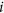, m^3^/kg; 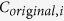 is the original consumption volume of product 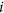, kg; 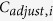 is the adjusted consumption volume of product 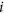, kg.

## Results

### Water footprint per weight unit of the crop and animal product

**Figure 2:**
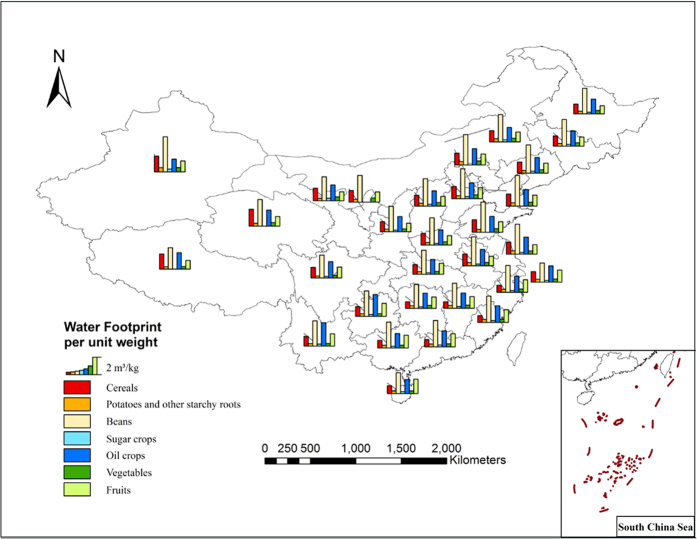
Geographical distribution of WF per unit weight of the main staple crops.

**Figure 3:**
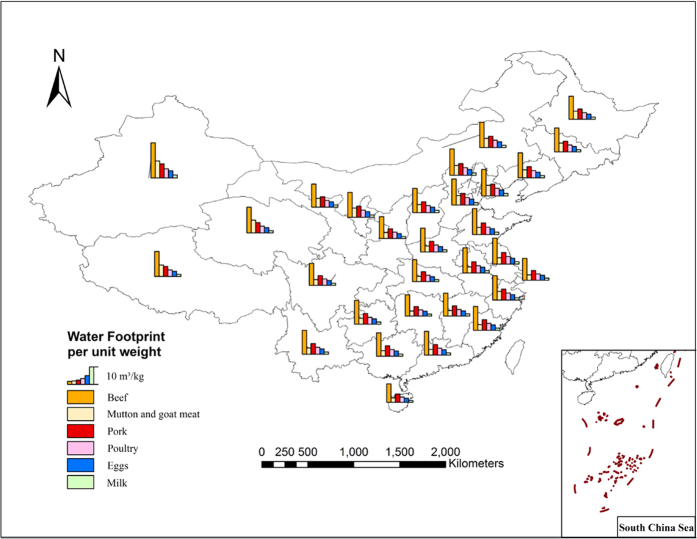
Geographical distribution of WF per unit weight of the main animal products.

The water footprint per weight unit of a crop and animal product reflects the amount water consumed (m^3^) during production of a single unit of mass (kg). This would indicate that if a relatively small amount of water was required to produce one kilogram of agricultural product, that product would have a small water footprint per unit weight, and a high level of water productivity. There are obvious differences between the total water footprint per weight unit in the 13 major food products analyzed. For crop product, the differences of the water footprint per weight unit between them is obvious, among which sugar crops having the lowest value at 0.21 m^3^/kg, and beans with the highest at 3.02 m^3^/kg. This is an extreme ratio of 14.38 between the two crops. Maize has the lowest total water footprint per weight unit among the cereals, which is 1.18 m^3^/kg. In general, oil producing crops and beans such as soybean and peanut have higher total water footprints per weight unit when compared with the cereals (Fig. 2). For animal product, due to large differences of growth cycles, feed structures and breeding patterns between them, there are distinct differences in water footprint per weight unit between them. In terms of the four meats, beef has the highest total water footprint per weight unit, which is 13.69 m^3^/kg, followed by pork (6.10 m^3^/kg), mutton(4.56 m^3^/kg) and poultry (3.97 m^3^/kg). The water footprint per weight unit of eggs is 3.09 m^3^/kg and that of milk is 1.28 m^3^/kg (Fig. 3).

The geographical distribution of the WF per weight unit of the 13 agricultural products is distinguished by the provincial and district boundaries of China, and it can be seen in Figs 2 and 3 that there is a relatively large difference in the distribution of the WF per weight unit of the agricultural products. For example, the spatial coefficient of variation of the WF per weight unit of the cereal products reached 0.25, the cereal crops (wheat, maize and rice) have lower WFs per weight unit in the Northeast China, Huang-Huai-Hai Region, several provinces along the middle and lower reaches of the Yangtze River and the south China province; while having higher values in part of the northwestern and southwestern provinces. The total WF per weight unit of potatoes differs from the cereals that its low values are in the Southwest, and in the middle and lower parts of Yangtze River, while the higher values are in the North and Northwest of China. For the animal products, the WF per weight unit of animal product in Northern region is generally greater than that in Southern region, which is mainly because feed crops, such as maize, bean and rice, have lower WF in Southern region, thus the indirect WF that came from the feed in the total WF of animal product is lower in Southern region. At the same time, Southern region has higher degree of centralized breeding than Northern region, consequently their utilization efficiency of water resources consumed in animal feeding process is higher. The above reasons lead to the “lower in the South, higher in the North” distribution pattern of the WF of animal product.

The conclusion that can be made from the comparison of the total WF per weight unit in the above crops is that the Northeast and Huang-Huai-Hai Region has an overall lower WF per weight unit which indicates a relatively higher water usage efficiency in these districts. Also, the regional differences of the WF per weight unit of the crop indicates that differences in the agro-climate and soil condition decide which types of crops are suitable for that area. From the perspective of WF composition, the proportion of green water in total WF in Southern region is higher than that in Northern region, which indicates that crop production in Northern region will consume more blue water resources. On the whole, WF of animal product is far greater than WF of crop product, the main reason behind this is that animal feeding will consume a large amount of crops, drinking water and production service water.

### Water footprint per calorie in crop and animal product

The main purpose of food products is to provide calories and nutrition, but depending on their physical and chemical properties, there can be large differences between the amount of calories and nutrition that each crop or animal product provides. Therefore, an analysis of water consumption during production of a calorie is a more practical method for determining the water footprint of staple agricultural products. The water footprint per calorie can provide a reference in devising the dietary guide which will require less investment of water resources while providing the same or more calories.

**Figure 4:**
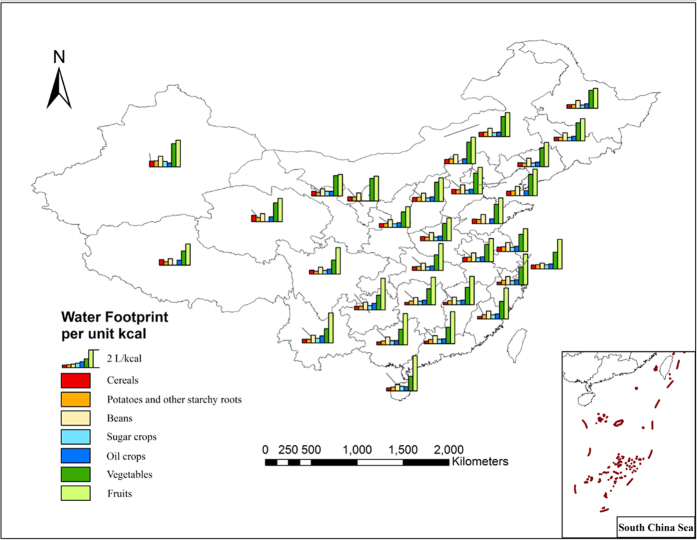
Geographical distribution of WF per unit calorie of the main staple crops.

The WF per calorie is determined by the WF per weight unit and amount of calories contained in each unit of weight. The results indicate that there are distinct differences in WF per calorie between the 13 analyzed staple crop and animal products. For the crops, the lowest WF per calorie is in potatoes, which is 0.43 L/kcal, followed by cereals (0.44 L/kcal), sugar crops (0.47 L/kcal), oil crops (0.54 L/kcal), vegetables (1.95 L/kcal) and fruits (3.04 L/kcal), with a ratio of 7.06 between potatoes and fruits (Fig. 4). When considering the calories provided by the crops per unit of water consumed, potato and cereal have higher water/energy conversion efficiency, while vegetables and fruits provide a relatively small amount of calories by using the same volume of water. The main reason for the differences in the WF per calorie between the crops is their physiological and biochemical differences. For example, maize is a C4 crop which has a higher photosynthetic conversion rate. Therefore, maize can convert more solar energy to bio-energy than other crops, under the same conditions, giving maize more calories per unit of water consumed. For animal product, pork has the lowest WF per calorie, which is 1.74 L/kcal, followed by milk (1.91 L/kcal), mutton (2.27 L/kcal), poultry (2.32 L/kcal) and beef (6.77 L/kcal), the extreme ratio reached 3.88. This indicated that to provide 1 kcal of energy, poultry needed to consume 2.32 L of water resources, while beef needed to consume 6.77 L of water resources. Therefore, the water/energy conversion efficiency of beef is relatively lower. In the dietary pattern, choosing poultry and eggs that have a lower WF per energy unit are more conducive to water savings.

**Figure 5:**
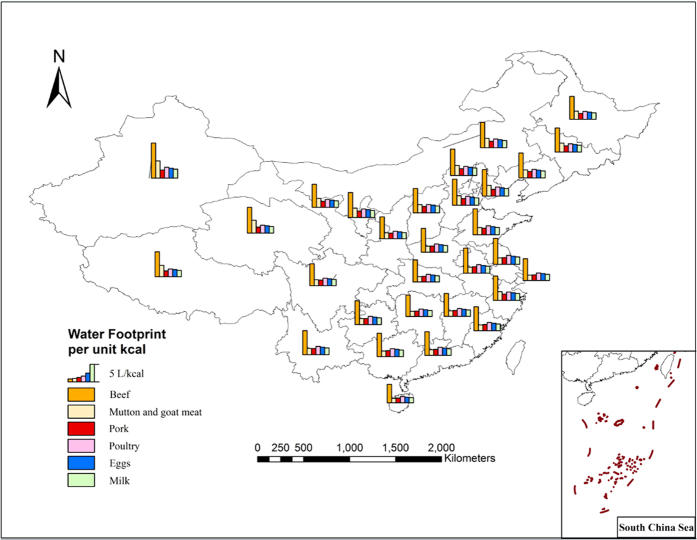
Geographical distribution of WF per unit calorie of the main animal products.

From the perspective of spatial distribution of WF per calorie of 13 crop and animal products, the crops with the lower WF per calorie are located in the Huang-Huai-Hai region, Northeastern regions, middle and lower reaches of Yangtze River and South China. This indicates that the crops from these areas require less water to provide each calorie, and thus they have high water/energy conversion efficiency. There is a higher level of WF per calorie in the Northwest, Southwest and partial provinces in southern China because these are arid and semi-arid regions which receive smaller amounts of precipitation and have higher evaporation rates. This leads to higher water consumption during crop growth production (wheat requires 50% more water in the XinJiang province than the AnHui provice). Additionally, some of these areas are located in hilly regions which limit the scale of farming operations, potentially leading to lower water/energy conversion efficiency. For animal product, water/energy conversion efficiency in Northern region is lower than that in Southern region. This is mainly because feed crops that have been consumed by animals, such as maize, bean and rice, have higher WF in Northern region, thus the indirect WF that came from the feed in total WF of animal product is higher in Northern region (Fig. 5).

### Analysis of the water savings through adjustments to the dietary pattern

Based on the fact that there are large differences between the WF per calorie of the 13 staple food products analyzed, it is theoretically possible to reduce the amount of water consumed during crop and animal production, while providing the same amount of dietary calories and nutrition by changing the crop consumption pattern. In order to calculate the water saving benefits through adjustments to the dietary consumption pattern, the study selected the consumption structure of Mainland China in 2010 as a benchmark, then two kinds of food consumption patterns were selected according to the Dietary Guidelines for Chinese Residents (CNS, 2013). One is a consumption pattern that is mainly based on crop products. In this scenario the consumption amount of animal products is depending on the lower limit of the dietary standard. The second scenario is a diet mainly based on animal products, where the consumption amount of animal products is dependent upon the upper limit of the dietary standard. By combining the consumption WF of food products under these two dietary scenarios, with the consumption WF of those 13 food products above for our base year, the effects of different dietary patterns on water resource consumption can be determined.

The results indicate that dietary pattern adjustments can bring obvious water savings while providing enough calories and nutrition as in the food guide based on CNS (2013). In scenario one, we have compressed the consumption of animal products that have a high WF, thereby reducing the consumption WF of meat products. The total amount of water savings will be 77.55 Gm^3^ in mainland China. Among them, green water is 59.79 Gm^3^, blue water is 9.19 Gm^3^ and grey water is 8.57 Gm^3^. In scenario two, we set up a dietary pattern mainly based on meat, and we have increased the consumption of animal products that have a high WF per energy unit. The results showed that water consumption increased 0.11 Gm^3^ under this dietary pattern than that in our base year (Table 1), which indicates that China’s overall food consumption structure in 2010 is close to the upper limit of meat consumption suggested by “Dietary Guidelines for Chinese Residents 2011”. Therefore, from the perspective of nutritional balance and water resource conservation, it is necessary to reduce the consumption of animal products.

**Figure 6:**
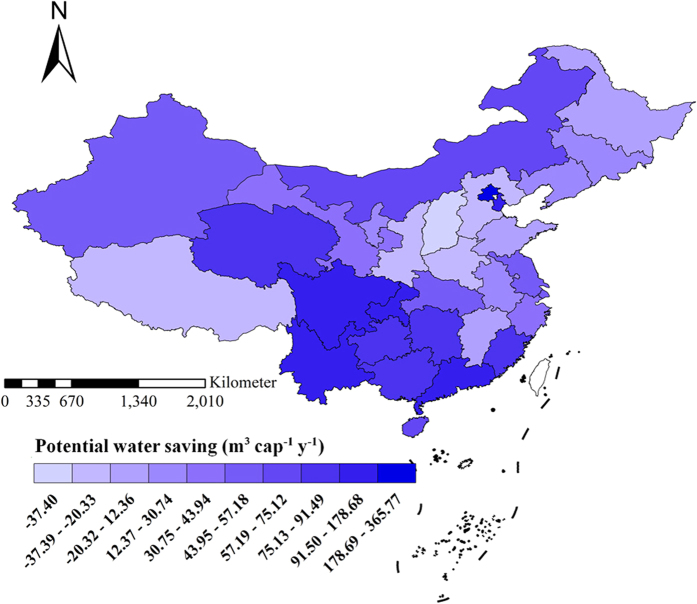
Per capital water saving potential through adjustments to the dietary pattern in each province in Mainland China.

In order to discuss the potential of the reduction of regional water consumption through the adjustment of dietary pattern in different regions, the study further analyzed food consumption WF per capita of each province in Mainland China under the baseline year and scenario one. By analyzing the difference between them we can reveal water saving potential of different regions through the adjustment of dietary pattern. The results showed that from the regional perspective, water saving potential per capita in Southern region is greater than that in Northern region, it is around 70 m^3^ cap^−1^ y^−1^ in many parts of Southern region. For example, it reached 178.68 m^3^ cap^−1^ y^−1^ in Guangdong Province. While water saving potential in Northern region is generally less than 30 cap^−1^ y^−1^, for example, it is −37.40 cap^−1^ y^−1^ in Shanxi Province, which indicates that the consumption structure in the base year in Shanxi Province has not yet reached the lower limit of meat intake that is recommended by the dietary pattern. From the perspective of regional social and economic development levels, economically developed regions have greater water saving potential per capita. For example, Beijing’s water saving potential per capita reached 365.77 cap^−1^ y^−1^, while that of Guangdong and Tianjin are 178.68 m^3^ cap^−1^ y^−1^ and 133.75 m^3^ cap^−1^ y^−1^ respectively. Economically under developed regions have a less water saving potential per capita (Fig. 6).

It can be seen that adjustments to the consumption pattern will lead to significant effects in agricultural water usage, and it can bring dramatic water savings while providing enough calories and nutrition for the resident. Especially for those economically developed regions, they can achieve greater water saving benefits by adjusting their dietary patterns.

## Discussion

**Figure 7:**
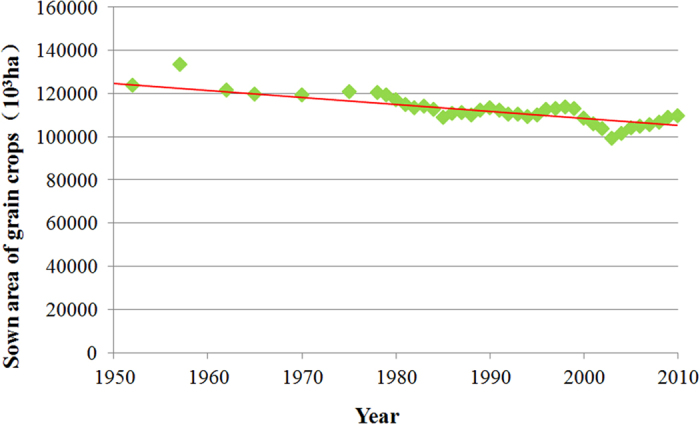
Trend of sown area of grain crops from 1950 to 2010 in Mainland China.

The increase in world population and food consumption will have a significant impact on demands for agricultural products and natural resources. Currently, nearly one billion people are estimated to be suffering from chronic malnourishment while global agricultural systems are concurrently degrading land, water, and biodiversity^30^. According to recent studies, unless there are dramatic changes in consumption patterns, food production needs to roughly double in order to keep pace with the projected population growth and the associated demand increase, and changes in the global dietary patterns^30^,^31^,^32^. Water and land are the essential resources for agricultural production, and in order to meet the demands of the global population expected by 2050, approximately 1 billion ha of uncultivated land will need to be converted to farmland^33^. However, most of the quality farmland has been already used for agricultural production, which means that further farmland expansion would occur on poor quality land that could not sustain high crop yields^34^. Meanwhile, with population growth and urbanized development, more and more cultivated land is being converted to cities and industrial areas, giving less room for future increases in cereal production. Figure 7 shows the downward trend in area planted with cereals in China between 1950 and 2010. This indicates a small possibility of improving cereal production by increasing the planted area.

**Figure 8:**
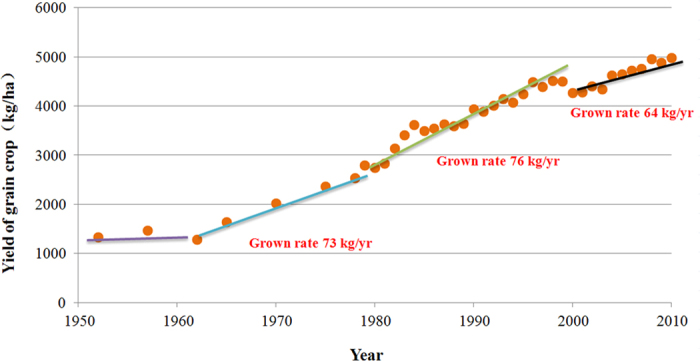
Trend of yield of grain crops from 1950 to 2010 in Mainland China.

Increasing cereal production without farmland expansion implies that we should increase the productivity of the existing farmland. The best places to improve crop yields may be on underperforming landscapes, where yields are currently below average^30^. However, the prospects for yield increases comparable to those of the past 40 years are unclear^35^. Figure 8 represents the grain production variation per ha per year, and phases of relatively fast increases from 1961 to 1980 and 1981 to 2000 can be seen (with the yearly rate of change 73 kg/yr and 76kg/yr). However, the increasing rate of grain production per ha has slowed in the past 10 years to rate of 64 kg/yr. This suggests that there is little room for increasing grain production through increases in yield of existing land.

In addition to land, water is also critical for cereal production. Irrigated land is only 15% of total cultivated land, but it produces approximately one-third of the world’s food^36^. Irrigated farmlands account for a substantial portion of increased yields obtained during the Green Revolution. Further agricultural production will require increases in irrigation, unless water use efficiency is substantially improved^37^,^38^. Water resources, however, are already limited in many areas and may be diverted to uses that compete with irrigation^39^. Moreover, water is regionally scarce. A band of countries, from China through India and Pakistan, and the Middle East to North Africa, either currently or will soon fail to have adequate water to maintain per capita food production from irrigated cropland^40^.

**Figure 9:**
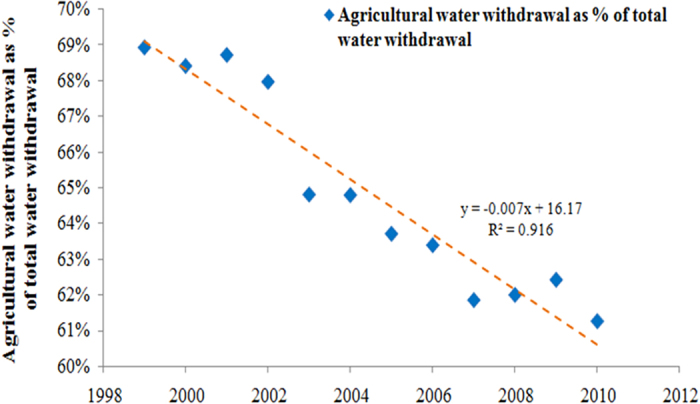
Agricultural water withdrawal as % of total water withdrawal in Mainland China.

Figure 9 shows the ratio of agricultural water usage with the total water usage in China from 1998 to 2010, and the agricultural water usage has gradually decreased because of the increase in industrial and domestic water usage. The amount of agriculture water usage will continue to decrease in the future because of population growth, increasing water usage in industry and urbanization, and climate change issues. Food production will, therefore, face several water shortage pressures in the future.

From 2003–2011, China increased its cereal production by about 32%^41^, largely by improving the performance of its least-efficient farms. Yet in the next two decades, 30–50% more food will still be needed to meet China’s projected demand^42^. China has little spare land, and water shortages are reaching critical levels in some areas. The limited water and land resources have become bottlenecks for guaranteeing food security in China. Meanwhile, the increase of cereal production could not rely on the increasing use of fertilizer, because excessive fertilization has been the cause of serious pollution^43^,^44^.

The results from the above analysis indicates that it is difficult to increase food production in China through expansion of the planting area or by investing in water technologies, due to the limited supply of cultivatable land and water supplies available to use. Additionally, increases to food production through yield increases on existing land also appear to be limited. The traditional water resource management is led by the analysis of supply and demand of the water resources, and the concept of water footprint provides a new index for analyzing the relationship between water resources and consumer demand. Traditional water resources management has the goal of improving the efficiency of water use, while water supply and demand management emphasizes water usage throughout the complete product supply chain, and the effect on water resources through trading and consumption models. Guiding the consumption pattern can effectively achieve the goal of water savings in agriculture besides improving the water productivity in crops through engineering or biotechnology from the production part while facing the challenges of population growth, water usage competition and climate change.

**Figure 10:**
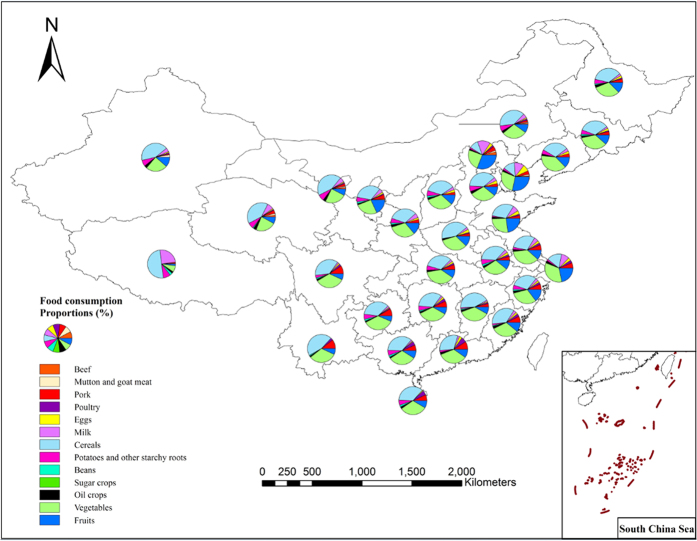
Food consumption pattern of each province in Mainland China.

The results from this case study indicated that promoting water saving consumption pattern will give a positive feedback on water consumption for agricultural production which provides a new solution for water saving in agriculture. This is to rationally combine the nutrient content in the food products and achieve the dual goal of dietary nutrition and water saving under the condition of guaranteeing the calorie and nutrition supply for human beings. At the same time, regional differences of consumption structures showed that the consumption proportion of animal products in Beijing, Shanghai, Guangzhou are at the forefront of China (Fig. 10), and these areas are also China’s economically developed regions, which indicates that these areas have a higher food consumption WF per capita, also indicating that these areas have a great potential to reduce the consumption of water resources by adjusting the dietary structure and increase the consumption of plant products.

But it is difficult to change the dietary pattern in a district in a short time because the dietary pattern is determined by the local culture, tradition, geography/climate, human and nature conditions. However, it is a necessary process to achieve the double goal of calorie supply and water saving by optimizing the consumption pattern from the consuming side in the near future because of the reality of water shortage and supply increasing day by day under the background of water supply limit. Meanwhile, virtual water strategy can be served as another way to alleviate regional water pressure. Countries or regions with scarce water resources could import water-intensive products to decrease the pressure on their own water resources. Meanwhile, the virtual water trade could save water globally if a water-intensive product is traded from a region where it is produced with high water productivity to a region with lower water productivity. Therefore, the virtual water flows process may prove significant for improving global water use efficiency and alleviating the pressure on local water resources. However, as the most populous country in the world, there is not enough food available for China to import. Since it may have important impacts on the global grain markets if China importing a large quantity of grain. So, the virtual water trade could alleviate China water crisis to some extent, but it is not able to solve the problem. Hubacek *et al.* (2009)^45^ explored the current trajectories and scenarios for urbanization and lifestyle changes and other important socio-economic trends in China by using Ecological and Water Footprints. The study indicated that the ability to construct sustainable communities in the future is a key challenge for China. Therefore, new approach is still needed to reduce water consumption and use the water in a more sustainable way. The adjustment of dietary pattern proves that it is potentially feasible to reach the objective of alleviating stress on water resources.

This study uses the concept of water footprint and extended its base by proposing the concept of WF per calorie provided by an agricultural product. Regional differences in WF per unit weight and calorie between districts and crops were discussed. The results of this article can serve as a framework to a water-saving dietary plan and a potential solution for future water shortages in grain production. Nevertheless, there are some limitations present in this study. Data unavailability and data uncertainty will cause uncertainty of the results of the study. Meanwhile, there are still some limitations in the scenario analysis of water savings under different dietary patterns, and owing to the scope of this study, we did not taking into account the bioenergy which has big influence on water consumption. Relatively insufficient data and methods renders this analysis as a first approximation, the present paper aims to provide an overview of the impact of changing crop consumption patterns on water consumption in China. Further study will be needed both for the value chain of food from production to consumption through trade mechanisms and uncertainties of the results.

## Conclusion

China faces two main challenges which are limited water resources and food safety. The traditional water resource management techniques focus on improving the efficiency of water usage through the production process. This study used the concepts of water footprint per unit weight and per calorie of crop and animal product based on the basic theory of water footprint. It also discussed the effect and level of significance of the production portion of water resource consumption through changing the dietary pattern of consumption. The study quantitatively compared the WF per unit weight and per calorie provided by each of the main crops and animal products produced in China and has the following conclusions:

There is a large difference among the WF per unit weight in different crop and animal products. For the crops, with a lower value in potatoes and tuber crops, and a higher value in beans and oil crops; also, geographically, the water footprint of the above crops is relatively low in the Northeast and South China while higher values are in the Northwest China. For the animal products, beef is with a higher WF per unit weight, while poultry has a relative lower value among the animal meat. Geographically, as a result of the difference between feed’s WF and breed type, WF per weight unit of animal product in Northern region is generally greater than that in Southern region.

There is a large difference among the 13 crop and animal products in water/energy conversion efficiency because of the physiology and biochemical character differences. Within the crops analyzed, cereals and potatoes have a lower WF per calorie and a higher percentage of solar energy converted to bioenergy, while consuming the same amount of water. For the animal products, beef has a higher WF per calorie, while that of poultry and eggs are lower, which indicates that beef has a lower water/energy conversion efficiency, while poultry and eggs have a higher one.

The scenario analysis of changing the dietary pattern indicates that adjustments to the consumption pattern will lead to significant effects in agricultural water usage, and it can bring dramatic water savings while providing enough calories and nutrition for the resident.

## Additional Information

**How to cite this article**: Sun, S. *et al.* Alleviating Pressure on Water Resources: A new approach could be attempted. *Sci. Rep.* **5**, 14006; doi: 10.1038/srep14006 (2015).

## Figures and Tables

**Table 1:** Analysis of the Water Savings under Different Adjustments to Consumption.

**Scenario**	**Green water saving (Gm^3^)**	**Grey water saving (Gm^3^)**	**Blue water saving in field scale (Gm^3^)**	**Total blue water saving (Gm^3^)**	**Total water saving (Gm^3^)**
High consumption share of crop products	59.79	8.57	4.64	9.19	77.55
High consumption share of animal products	−1.27	0.98	0.09	0.18	-0.11

Note: positive numbers represent water saving condition compared with the value from 2010; negative numbers represent more water consumption compared with 2010.
